# The Limbal Niche and Regenerative Strategies

**DOI:** 10.3390/vision5040043

**Published:** 2021-09-22

**Authors:** Sohil Amin, Elmira Jalilian, Eitan Katz, Charlie Frank, Ghasem Yazdanpanah, Victor H. Guaiquil, Mark I. Rosenblatt, Ali R. Djalilian

**Affiliations:** 1 Department of Ophthalmology and Visual Sciences, University of Illinois Chicago, Chicago, IL 60612, USA; sohila2@uic.edu (S.A.); jalilian@uic.edu (E.J.); ekatz8@uic.edu (E.K.); cfrank27@uic.edu (C.F.); ghyazdan@uic.edu (G.Y.); vguaiqui@uic.edu (V.H.G.); mrosenbl@uic.edu (M.I.R.); 2 Richard and Loan Hill Department of Bioengineering, University of Illinois Chicago, Chicago, IL 60612, USA

**Keywords:** ocular surface regeneration, limbal epithelial stem cells, limbal stem cell niche, corneal epithelium, mesenchymal stem cells

## Abstract

The protective function and transparency provided by the corneal epithelium are dependent on and maintained by the regenerative capacity of limbal epithelial stem cells (LESCs). These LESCs are supported by the limbal niche, a specialized microenvironment consisting of cellular and non-cellular components. Disruption of the limbal niche, primarily from injuries or inflammatory processes, can negatively impact the regenerative ability of LESCs. Limbal stem cell deficiency (LSCD) directly hampers the regenerative ability of the corneal epithelium and allows the conjunctival epithelium to invade the cornea, which results in severe visual impairment. Treatment involves restoring the LESC population and functionality; however, few clinically practiced therapies currently exist. This review outlines the current understanding of the limbal niche, its pathology and the emerging approaches targeted at restoring the limbal niche. Most emerging approaches are in developmental phases but show promise for treating LSCD and accelerating corneal regeneration. Specifically, we examine cell-based therapies, bio-active extracellular matrices and soluble factor therapies in considerable depth.

## 1. Corneal and Limbal Epithelium

**Figure 1:**
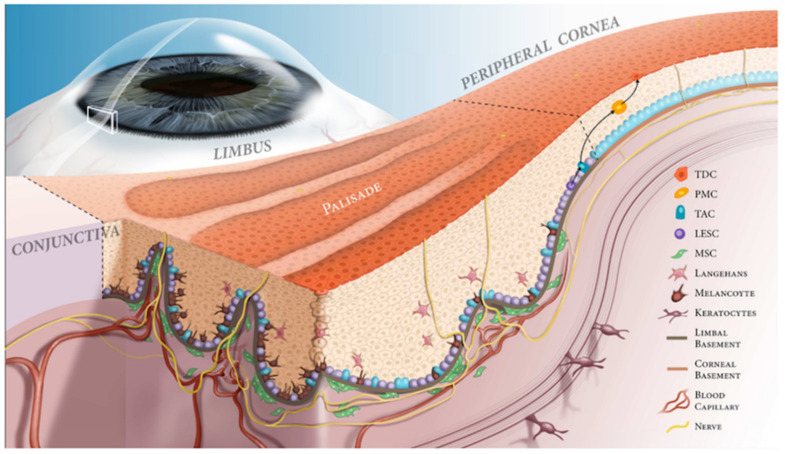
Limbal niche. Illustration of the limbal niche, focusing on the palisade of Vogt. The palisades of Vogt form crypts in the limbal epithelium, allowing for close contact between LESCs and supportive cells, including melanocytes, keratocytes, mesenchymal stem cells and Langerhans cells. These cells, along with the basement membrane and neurovasculature, provide growth factors, nutrients and structural support to promote proper LESC proliferation and differentiation. At the border of the limbal and corneal basement membranes, LESCs divide into progenitor cells or transient amplifying cells (TAC). The TACs divide into postmitotic cells (PMCs) and migrate centrally. These PMCs differentiate into terminally differentiated epithelial cells (TDCs) to replace lost cells on the corneal surface. Use of illustration permitted by [5].

The corneal epithelium has an essential role in maintaining corneal transparency and providing a protective barrier. Due to the high cell turnover of this layer, these epithelial cells are constantly replenished by limbal epithelial stem cells (LESC). These specialized stem cells are supported by the limbal niche (microenvironment), which is located at the periphery of the cornea bordering the conjunctiva. The limbus houses LESCs and contains unique cellular and chemical characteristics that promote growth and differentiation of LESCs. Molecular analysis has shown that the limbus is organized differently than the rest of the corneal surface, in an important anatomical pattern known as the palisades of Vogt [1,2]. The palisades of Vogt are undulations in the stroma and Bowman’s membrane where the epithelium extends deeper and corresponds to the limbal niche. Many cell types are present in the limbus, including melanocytes, immune cells, stromal cells, nerve cells and vascular endothelial cells (Figure 1) [3,4].

**Figure 2:**
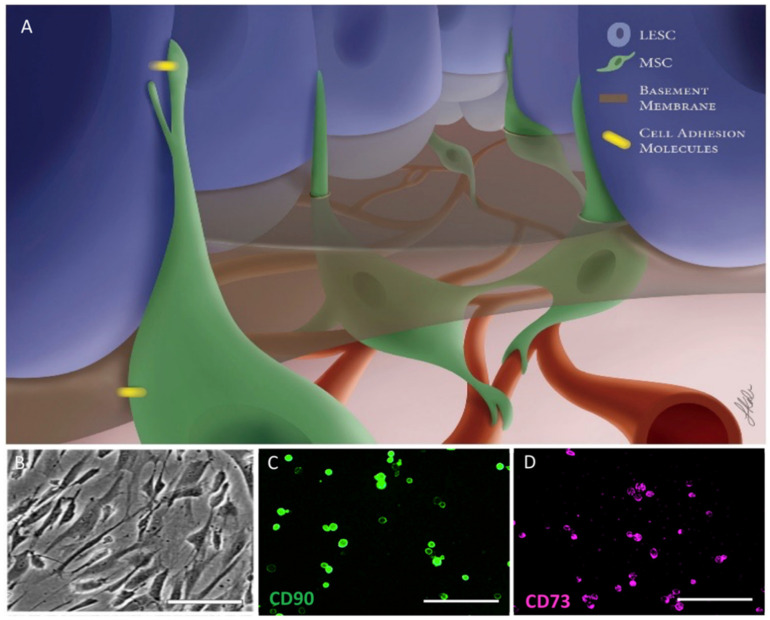
Limbal stem cell interactions. (**A**) Illustration demonstrating the interaction between limbal MSCs and LESCs. Use of illustration permitted by [5]. (**B**) Representative picture of human Limbus MSCs using brightfield microscopy. (**C**,**D**) Human limbus mesenchymal stromal cell in suspension expressing markers CD 90 and CD73.

Two notable components of the limbal niche are the stroma and mesenchymal stromal cells (MSCs). MSCs have been found in the human limbus underlying the basal epithelium and have important interactions with the LESCs (Figure 2B–D) [6]. Signaling pathways, intracellular contact and cytokine expression from MSCs are critical to LESC functioning [7,8].

## 2. Pathology of Limbal Niche

Traumatic, genetic, or immunologic disturbances to the cornea can disrupt the limbal niche microenvironment, resulting in limbal stem cell deficiency (LSCD). Under pathological conditions, the damaged cornea can have persistently increased levels of cytokines and other inflammatory mediators, such as interferon-γ, IL-1α, IL-1β, IL-6 and vascular endothelial growth factor (VEGF), which disrupt the conditions needed for the LESCs to proliferate [9,10]. This inflammatory milieu leads to recruitment of T-lymphocytes, neutrophils and macrophages to the site of injury, which can disrupt the LESC niche [11]. Additionally, inflammation can cause pathological changes in the extracellular matrix of the limbus, such as increased vascular and lymphatic vessel formation [12]. This leads to abnormal density and morphology of LESCs through decreased extra cellular matrix (ECM) adhesiveness and altered expression of stem cell markers [12,13]. Possible causes of inflammation to the limbus in humans can include bacterial infection, viral infection, hypersensitivity response and traumatic injuries [12].

The net effect of inflammatory injury is an altered limbal environment that can no longer support LESCs, causing reduction in the colony-forming capability of LESCs [13]. Consequently, the regenerative and anti-angiogenic ability of the corneal surface is severely hampered. Pathological fibrosis, vascularization and abnormal goblet cell deposition occur, and the cornea becomes covered with an opaque and vascularized epithelium, which promotes scarring. Clinically, this is described as conjunctivalization, which significantly impairs the transparency of the cornea and leads to loss of vision.

To avoid conjunctivalization of the cornea from LSCD, the LESC population as well as the limbal niche must be restored. The microenvironment is crucial to the rehabilitation of the proliferative capability of LESCs. Successful treatment should restore LESC functionality in addition to resolving the underlying dysfunction causing the LSCD. Strategies for repairing LESC function are increasing, with key and emerging methods detailed below. While many of the strategies are just beginning to reach the clinic, they represent promising advancements in restoring the limbal niche.

## 3. Therapeutic Regeneration of the Limbal Niche

The goal of LSCD therapy is to restore the functionality and population of LESCs, both through increasing cell numbers and repairing the limbal niche. In milder cases, stopping the underlying traumatic, immunologic, or chemical source of injury may be sufficient, allowing for natural repopulation of epithelial cells [14,15]. Other methods, including improving the tear film through autologous serum tears and protecting the corneal epithelium with scleral contact lenses, have shown promising results in reducing pain and enhancing vision [16]. Contact lenses have also been used as novel drug delivery devices [17]. These strategies are aimed at restoring the function of existing epithelial and stem cells to accelerate corneal healing [18].

When inflammation is concurrent with LESC damage, and especially when it is the cause of ocular surface disease, anti-inflammatory therapy is crucial in promoting rehabilitation. Topical corticosteroids are a mainstay of treatment for autoimmune surface disease and pro-inflammatory injuries such as chemical burns to slow or prevent permanent damage to the limbal niche [19]. Other topical anti-inflammatory agents, such as cyclosporine, tacrolimus and lifitegrast, can also be employed when inflammation is a result of a chronic condition, such as dry eye syndrome, or atopic or vernal conjunctivitis. Systemic conditions such as mucous membrane pemphigoid often require treatment with systemic immunomodulatory therapy and should be controlled as best as possible before proceeding with surgery [19]. Importantly, immunosuppression is also a key facet of management after surgical transplantation with an allograft.

In the case of severe LSCD or other severe corneal disease, reconstruction/transplantation of the limbus along with other epithelia is needed [20]. These interventions are best used early in the disease process, as an increasingly progressive inflammatory environment in the limbus leads to permanent loss of the limbal niche, making visual rehabilitation difficult [15,21]. Other emerging methods below aim to augment the transplantation process or provide an alternative therapeutic option.

### 3.1. Cell-Based Therapies

#### 3.1.1. Limbal Transplantation

Limbal transplantation is a procedure where limbal tissue is surgically transplanted to an ocular surface with LSCD. The limbal niche and LESCs are transplanted simultaneously. All procedures should be accompanied by anti-inflammatory therapy [22]. This approach is suitable for patients with severe/total LSCD. In unilateral cases of LSCD, conjunctival limbal autograft (CLAU) from the unaffected eye is the standard procedure [14,23,24]. An emerging technique is simple limbal epithelial transplantation (SLET), where a 4 mm^2^ area of limbal tissue is taken from the donor eye, reduced to smaller pieces and grafted onto the diseased cornea with an amniotic membrane scaffold [25,26,27]. This technique reduces the risk of injury to the donor eye, with promising results thus far. For bilateral severe/total LSCD, allogeneic limbal grafts from healthy human or cadaver donors are primarily used. As the grafts are allogeneic, long-term immunosuppression is needed for these patients. The overall success rate is variable, ranging from to 30% to 80% in different studies, and is influenced by factors such as cadaver vs. live donor tissue, disease etiology and immune matching [28]. Long-term studies have shown that in allografts, both donor and host epithelial progenitor cells are observed on the ocular surface [28]. This suggests regeneration of the limbal niche due to the presence of host epithelial progenitor cells, most likely coming from reactivated host LESCs. This further strengthens the idea that revitalizing the host limbal niche leads to host LESC reactivation, facilitating repopulation of the corneal epithelium.

#### 3.1.2. Ex-Vivo Epithelial Cell Cultivation

In cultivated limbal epithelial transplantation (CLET), a small portion of limbal tissue is harvested from the donor eye and expanded ex vivo in culture [29,30]. Since the area of harvested tissue is smaller than in traditional CLAU, injury to the donor eye is minimized, while the benefit of reduced immune reaction compared to allograft is maintained [28,31]. Similar to CLAU, CLET is thought to facilitate limbal niche restoration through both repopulation of the microenvironment with LESCs as well as signaling to the area of injury to promote host tissue healing [32]. ECM components and growth factor components may be used to provide an environment similar to the limbus for the continued proliferation of LESCs ex vivo. Culture systems including MSCs and limbal fibroblasts have also been used [33,34]. Human-derived serum and growth factors have shown reduced risk in transmitting animal-derived infections [35]. Once the cells have expanded in culture, the LESCs are transplanted within a scaffold. The most common scaffold used in patients is human amniotic membrane and fibrin gel [36,37,38,39]. In one study, autologous stem cells cultivated on fibrin substrate achieved successful regeneration of the corneal surface in 14 out of 18 patients with LCSD [33]. Other scaffolds have been explored, including collagen hydrogels, fibrin and siloxane contact lenses, which are used to improve transparency and reduce risk of infection [40,41,42,43]. Improvements in the formulation of scaffolds could lead to increased use of ex vivo cell cultivation for ocular surface rehabilitation.

In cases of bilateral LSCD where ex vivo autologous limbal epithelial cell cultivation is not possible, other autologous cell sources have been explored. One option is autologous conjunctival epithelial cells. Studies have been conducted using ex vivo non-keratinized conjunctival epithelial cells cultured on human amniotic membrane [44,45,46]. Another option is using cultivated oral mucosal epithelial transplantation (COMET), which has been shown stabilize the ocular surface and achieve similar visual acuity outcomes to those of CLET [47,48,49]. While these sources may provide some stability to the ocular surface, neither is able to restore a limbal niche and a corneal phenotype.

Most recently, induced pluripotent stem cells (iPSCs) and human embryonic stem cells (hESCs) have been differentiated to limbal/corneal epithelial cells with promising results [50,51].

#### 3.1.3. Mesenchymal Stromal/Stem Cells

Mesenchymal stromal/stem cells (MSCs) are multipotent stem cells that can be found in the bone marrow, fat, limbus and many other tissues. MSCs have gained attention for their potential use in the rehabilitation of the limbal niche [52]. MSCs support local stem cells in replacing damaged cells as well as secreting anti-inflammatory cytokines, leading to immunosuppression. They also have the ability to produce their own ECM in 3D culture systems [53].

While MSCs cannot directly replace corneal epithelial cells or LESCs, they have promising functionality in restoring the limbal niche. Multiple animal model studies have shown improvements in LSCD, chemical burns and dry-eye syndrome after MSC transplant (MSCT) [54,55,56]. One example used bone-marrow-derived MSCs with a hydrogel in an ocular surface burn rat model [57]. This led to enhanced healing of the corneal epithelium with better transparency, less vascularization and enhanced anti-inflammatory activity when compared to controls. MSCs have been shown to be effective in mice when administered locally via subconjunctival injection but can also be given intravenously [58].

Since MSCs have been observed in the limbus in vivo, corneal-specific lines of MSCs (L-MSCs) could provide additional therapeutic benefit. These L-MSCs have direct contact with LESCs in the limbal niche as well as exchanging various signaling molecules [59,60]. L-MSCs show similar anti-inflammatory effects to those of bone-marrow-derived MSCs, and they provide additional anti-angiogenic activity by producing soluble factors such as fms-like tyrosine kinase-1 (sFLT-1) [61]. Bone marrow MSCs also demonstrate some anti-angiogenic activity but do not secrete sFLT-1. However, bone marrow MSCs are able to differentiate into L-MSCs, which can then be transplanted for therapeutic use. One pilot study showed similar rates of success between CLET and autologous bone marrow MSC transplantation for LSCD: 76.5–85.7% of 18 eyes with LSCD achieved restoration of the corneal surface after 6-12 months [62]. There are no data to support the use of MSCT in combination with CLET or CLAU. However, as MSCs and LESCs both play crucial roles in the limbal niche, it is plausible that transplantation of both cell types could have a synergistic effect. In addition, administration of L-MSCs with an ECM could be an important future approach for moderate-to-severe ocular surface pathologies.

#### 3.1.4. Melanocytes

Melanocytes are found in the limbal area, disappearing closer to the corneal periphery so as not to interfere with the corneal epithelium [63]. Their function is the production of melanin to provide limbal stem cells with protection against UV radiation, as well as possible secondary functions, including free radical scavenging and immunological support [63,64]. Melanocytes in the limbus have direct contact with LESCs via cadherins and L1CAM [64]. This suggests they play a role in supporting LESCs in the limbal niche. One study examined the effect of limbal melanocytes on LESCs in 2D and 3D cultures. They observed that the melanocytes co-localized with epithelial cells in native limbal crypts. The 3D culture showed enhanced development of epithelial sheets with increased differentiation compared to the 2D culture. They concluded that melanocytes could play in important role in maintaining the limbal niche [65]. Another study has shown that melanocytes can inhibit T cells and vascular endothelial cells, helpful in regenerating the corneal surface [13]. The role of melanocytes in the cornea is not yet completely understood, and further studies are required to determine their potential therapeutic applications in the limbal environment.

### 3.2. Biologically Stimulating Scaffolds

With the goal of restoring the limbal niche in mind, scaffolds have been developed to mimic the niche microenvironment and facilitate successful stem cell proliferation. As laid out above, the limbal niche has a unique topography and composition that are specifically suited to LESC development. These factors must be considered when developing and evaluating new scaffolds. One other factor that may be relevant is corneal stiffness. The importance of the characteristic of corneal and limbal tissue has been recognized in the pathophysiology of diseases such as keratoconus and, more recently, in glaucoma [66,67]. Interestingly, corneal stiffness may also play a role in LSCD, as it has been shown to be associated with cell signaling pathways in wound healing and fibrosis [68]. Specifically, TGF-beta and latrunculin B appear to mediate the corneal scarring response, with a higher degree myofibroblast activation on stiffer substrates [69]. These biomechanical and structural features are likely directly relevant to the efficacy of various biologically stimulating scaffolds.

#### 3.2.1. Human Amniotic Membrane

The human amniotic membrane is the most frequently used scaffold as therapy for ocular surface disorders [70]. The amniotic membrane is the innermost layer of the placental membrane surrounding the fetus, which forms the amniotic cavity and is obtained by peeling off the fetal membranes [71]. It is particularly useful for ocular applications given its lack of vascularization and innervation as well as decent transparency [72]. Additionally, the ECM of the amniotic membrane contains collagen, fibronectin and growth factors (epidermal growth factor and hepatocyte growth factor) that stimulate natural regeneration of the ocular surface [73]. The collagen-rich structure serves as a useful scaffold for the delivery of various cells, such as LESCs, to the ocular surface [74]. While it has been the standard scaffold for many years, amniotic membranes have shortcomings as a delivery scaffold to the corneal surface. First, amniotic membranes are somewhat opaque and have a small risk of carrying infectious diseases [5]. Additionally, amniotic membranes have low tensile strength, with much variability between batches. Most importantly, the benefits of amniotic membranes are temporary due to gradual graft erosion after transplantation. Therefore, therapy with an amniotic membrane may not reconstruct the limbal niche in the long term. Due to these drawbacks, other cell delivery scaffolds are being explored.

#### 3.2.2. Fabricated ECMs

While replenishing cell numbers in cases of LSCD is important, the ECM environment of the limbal niche is also essential for the promotion of sustainable growth and differentiation of LESCs. Fabricated ECMs not only provide a stable cell delivery platform, but they can mimic the limbal ECM. One such fabricated ECM is limbal crypts using chiefly type I collagen, simulating the palisades of Vogt in the limbus. These crypts are 3D printed using type I collagen, elastin and laminin to replicate the complex structure and alignment of the limbal environment [75]. With appropriate structure and composition, they support proliferation and appropriate the differentiation of human LESCs in vivo.

Another promising new approach for ECM scaffolds, particularly for stroma regeneration, is the use of decellularized porcine or human corneas [5,76]. This approach utilizes efficient decellularization of the corneas to remove cells and immunogenic antigens while preserving the structure and functionality of the ECM. Many structural proteins and various healing factors are also preserved. Several different methods are used for decellularization, including detergents, freeze thawing, osmotic solutions and ribonucleases to remove antigenicity to the host [77,78]. Decellularized corneas have been shown to support cultivation of corneal epithelial cells and have been successfully transplanted into animal models [79,80]. Our group previously studied decellularization of human cadaver corneas with hypertonic NaCl, followed by nuclease treatment and subsequent transplantation into a limbal injury rat model. The results demonstrate supported growth of epithelial cells as well as inhibited corneal haze [81].

#### 3.2.3. Bio-Active Hydrogels

Another approach utilizing decellularization is the fabrication of bio-active hydrogels. These hydrogels are derived from digested decellularized corneas [76,82]. In the studies by our group, we have fabricated a thermoresponsive hydrogel from a decellularized porcine cornea extracellular matrix (COMatrix, Cornea Matrix) via digestion with pepsin/HCl [82]. The COMatrix has been characterized biomechanically and also compositionally using mass spectrometry. COMatrix is rich in proteins with corneal epithelial wound-healing effects, such as lumican and keratocan. This bio-active hydrogel is compatible with both epithelial and stromal cells, resulting in promising cell delivery vehicles for three-dimensional structures [42]. Moreover, in vitro studies have shown a proliferative effect of COMatrix on human corneal epithelial cells as well as enhancing corneal epithelial wound healing in in vivo animal models [76].

The proteins and healing factors, including collagen, hyaluronic acid, elastin, glycosaminoglycan and more, can support the restoration of the stroma and limbus. Other approaches to a bio-active hydrogel include a collagen-coupled polymer hydrogel that supports epithelial wound closure [83,84], a collagen hydrogel with cross-linking in situ designed as a corneal stromal substitute [85] and a silk-film-derived hydrogel that could be patterned to change corneal epithelial gene expression on the basis of structure [86].

#### 3.2.4. Biomaterials for Construction of Scaffolds

With regard to natural or synthetic scaffolds used for ECMs, hydrogels and stem cell cultivation, a variety of biodegradable materials can be used. Natural options include those listed above, such as the human amniotic membrane or decellularized porcine/human cadaver extracellular matrix. The advantages of natural scaffolds include a high degree of biodegradability, natural healing factors and good optical transparency [87]. Other options for construction of scaffolds include fibrin, collagen and constructed polymers. Fibrin membranes are chiefly constructed with fibrinogen and thrombin, and they have a documented history of use in ophthalmology as a sealant [88,89]. The advantages of fibrin include ease of preparation and established success in trials for LSCD [43]. Collagen as a scaffold is well established in tissue engineering for cell transplantation and can be of natural or synthetic origin. Type I collagen is the most used for the cornea and can be modified in many ways, such as cross-linking or plastic compression, to improve mechanical strength and resistance to degradation [90]. The use of collagen is advantageous for its biocompatibility, mechanical strength (cross-linked) and high availability [87]. Synthetic polymers for ophthalmology include polymethacrylate and polyethylene glycol, both of which have shown support for LESC cultivation in models but have not been trialed in humans [91,92]. They can be used as scaffold bases or modified onto other compounds, such as type I collagen. The advantages of synthetic polymer scaffolds include easy mass-production, manipulability and chemical stability [87].

### 3.3. Therapeutic Factor-Derived Solutions

#### 3.3.1. Blood Product Derivatives

While many of the complex interactions and signaling molecules of the limbal niche are not fully understood, it is clear that proper signaling and growth factors are essential for growth and proliferation of LESCs and corneal epithelial cells. Administration of soluble factors is effective at restoring function to a disrupted limbal microenvironment. One of these methods uses blood-derived factors, which are increasingly popular in the clinical setting of corneal surface diseases. Currently, autologous/allogeneic serum eye-drops (ASEs) and platelet-derived solutions are used, as they are abundant in cytokines, growth factors and vitamins that are normally required for corneal epithelial proliferation and differentiation [93]. ASEs have been shown to contain epidermal growth factor, fibronectin, TGF-β and other cytokines important for corneal epithelial and LESC homeostasis. Thus, they show effectiveness in diseases involving the corneal surface, such as in graft-versus-host-disease (GVHD), Sjögren syndrome, conjunctivitis, neurotrophic keratitis and dry eye disease, in which they reduce symptoms and increase patient satisfaction [94,95,96]. The drawbacks of ASEs are increased risk of infections, limited manufacturing reliability and imperfect stability [97]. In addition, the use of ASEs may be limited in those with systemic inflammatory conditions due to the difference in key serum factors [98,99].

Currently, three types of platelet formulations are used, including platelet releasate (PR), platelet-rich plasma (PRP) and plasma rich in growth factors (PRGF). They are obtained from the supernatant of anti-coagulated whole blood [100]. These therapies are potentially useful for the regeneration of the limbal niche due to the abundance of growth factors, such as epidermal growth factor, TGF-β, IGF-1, PEDF and bFGF-2 [101]. Various in vivo and clinical studies have shown reconstructive and regenerative ability, such as in dry eye disease, although efficacy differs depending on formulation and preparation methods [102,103,104].

#### 3.3.2. Amniotic Membrane Derivatives

As detailed earlier, the human amniotic membrane stimulates the regeneration of the ocular surface due to the presence of many growth factors. These factors have been collected from the human amniotic membrane supernatant following centrifugation, resulting in a cocktail of soluble factors. This has been formulated as an eye drop called amniotic membrane extract eye drop (AMEED) [105]. AMEED has shown beneficial effects in enhancing in vivo cultivation of LESCs for patients with LSCD [105]. However, AMEED has yet to be proven effective in a clinical setting. Further purification from the amniotic membrane factors yields HC-HA/PTX3, which has been identified as a factor useful in regeneration of the limbal microenvironment. It is known for its potential anti-inflammatory and anti-scarring actions [106]. A study using a mouse model of GVHD dry eye disease showed that injection of HC-HA/PTX3 led to significantly reduced inflammatory cell infiltration and increased LESC proliferation via the Wnt/BMP signaling pathway [107].

#### 3.3.3. Growth Factor Formulations/Cell Secretions

In addition to blood product and amniotic membrane derivatives, there are other specific soluble growth factors that can be used as therapy for ocular surface diseases by modulating inflammation, reducing vascularization and increasing limbal niche signaling.

Nerve growth factor (NGF) has a critical role in developing and maintaining the ocular surface and vision [108]. Normally secreted in the aqueous humor, NGF modulates ocular surface healing in animal and clinical models for severe corneal diseases [109]. A recombinant NGF, rhNGF, was recently approved by the FDA for the treatment of neurotrophic keratitis, demonstrating the potential of topical NGF for the treatment of certain ocular surface diseases [110].

Another growth factor that shows therapeutic efficacy in the ocular surface is pigment epithelium-derived factor (PEDF). PEDF promotes stem cell survival and maintenance of multi-potency, including LESCs in vitro [111]. An animal model showed that during limbal transplantation, PEDF promoted proliferation of LESCs compared to control, enhancing the restoration of the limbus and its function [112]. Further clinical measurements are needed to prove efficacy in humans. Other factors that play key roles in corneal wound healing include fibroblast growth factor (FGF), keratinocyte growth factor, ciliary neurotrophic factor, interleukin (IL)-1 and hepatocyte growth factor [112].

Due to the difficulty of producing individual recombinant factors, conditioned media have been developed from cultivated cells that contain a cocktail of many growth factors. Secretomes are made from the supernatant of in vitro cultivated cells, similar to AMEED. Of particular importance are secretions from mesenchymal stem cells, as the media contain a wide variety of growth factors useful in restoring the corneal surface [113]. Human bone-marrow-derived MSCs contain IL-1, IGF-1, stem cell factor, TGF-β, FGF-2 and more in high amounts, which have proven useful for restoring the limbal niche [114]. Additionally, studies have shown that MSC secretomes increase the expression of the CD44 receptor to enhance the binding of hyaluronic acid [108]. This process facilitates wound healing and minimizes scar formation through mechanical interactions [115].

**Figure 3:**
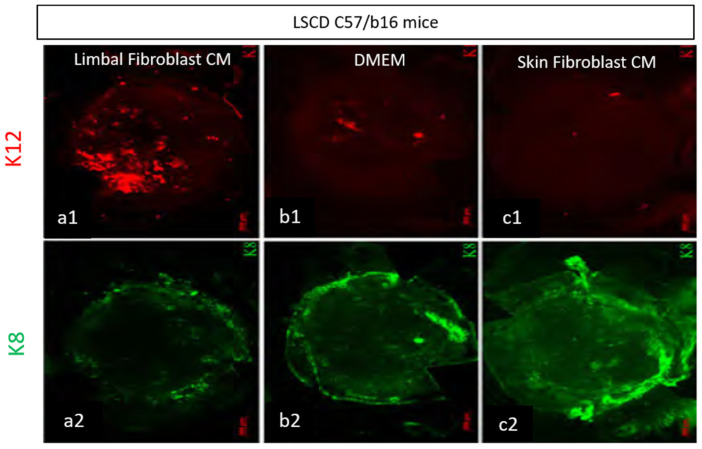
Representative immune staining of whole-mount corneas from mouse model of LSCD that are treated with three conditioned media. Corneas treated with human limbal fibroblast-conditioned media showed consistent expression of K12 (red, (**a1**)) and lower expression of K8 (green, (**a2**)) which shows the therapeutic effect of this media. However, corneas treated with DMEM or human skin-conditioned media as a negative control illustrated low expression of K12 ((**b1**,**c1**) respectively) and high expression of K8 (green, (**b2**,**c2**) respectively). Mouse model of LSCD treated with conditioned media. This study created a mouse model of LSCD through limbus to limbus scraping. Mice were then treated with three weeks of limbal fibroblast-conditioned media (A–F), DMEM (G–I) or skin-conditioned media (J–L). K8-positive cells fluoresce green, while K12 positive cells fluoresce red. M and N are magnified and stained with DAPI [55].

Additionally, conditioned media from limbal fibroblasts have shown therapeutic benefits. Limbal fibroblasts regulate differentiation and maintenance in the corneal epithelium and limbus, including LESCs. In one study, Amirjamishidi et al. created a mouse model of LSCD in C57/b16 mice [116]. The mouse model was given LSCD by scraping of the entire cornea from limbus to limbus to perform an epithelial debridement. Then, the authors treated these LSCD corneas with three different media, including conditioned media derived from human limbal fibroblast cultures, and two other media (Dulbecco’s serum-free medium (DMEM) and skin fibroblast conditioned media) as a negative control. Skin fibroblast cultures were initiated from fresh human foreskin tissue. Over several steps and removing the epidermis, the dermis was cut into smaller pieces, which were used as explants to initiate dermal fibroblast cultures. Mice were treated topically with these media for up to 3 weeks. Mice treated with limbal fibroblast-conditioned media revealed considerable growth of corneal-type epithelial cells shown by expression of K12 on the corneal surface shown (Figure 3(a1)) and less conjunctival goblet cells shown by fewer expression of K8 (Figure 3(a2)). However, the LSCD corneas treated with DMEM and skin fibroblast condition media were found to be covered primarily by conjunctival type epithelium, low expression of K12 (Figure 3(b1,c1)) and high expression of goblet K8 cells (Figure 3(b2,c2)). This study showed that cell culture media conditioned by limbal fibroblast cells (and not the cells themselves) appear to contain factors that are therapeutically beneficial in a model of limbal stem cell deficiency and can significantly improve growth in the corneal epithelium, allowing for the proposal of a new, non-invasive approach in the treatment of limbal stem cell deficiency [55].

As limbal mesenchymal stem cells best match the environment of the limbal niche, it is logical that conditioned media from L-MSCs can be used to accelerate epithelial wound healing. Secretomes from L-MSCs show increased epithelial wound healing, reduced angiogenesis and diminished ability of macrophages to cause corneal vascularization [117].

#### 3.3.4. Exosomes

Another emerging therapy that utilizes cellular secretions is MSC-derived exosomes. Exosomes are endosome-derived extracellular vesicles secreted by a variety of cells, ranging in size from 40 to 160 nm [118]. Their contents include proteins, lipids and nucleic acids, which serve to induce signaling responses in cells that uptake exosomes [119,120,121]. The signaling properties of exosomes are highly variable depending on the cell of origin. As exosomes are secreted along with many other particles, they require isolation from other products. The most commonly used method is ultracentrifugation, which is the easiest and most cost-effective method for isolating large amounts of exosomes [122]. For smaller volumes, immunoaffinity chromatography can be used to increase the purity of exosomes that have been isolated [123]. Other methods for separation include ultrafiltration, size exclusion chromatography and precipitation [124].

**Figure 4:**
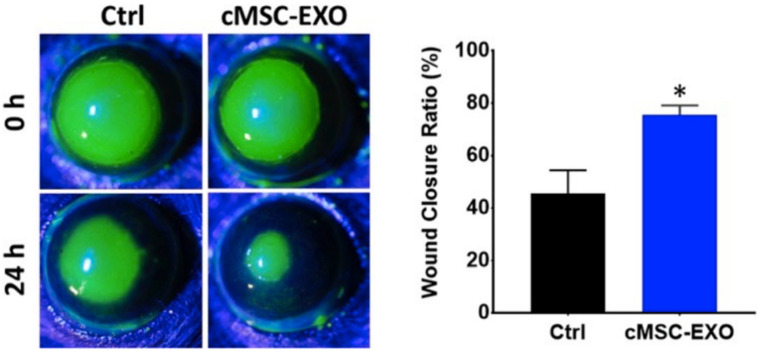
Exosomes from cornea MSCs promote corneal epithelial wound healing. In vivo images using cornea MSC-derived exosomes in corneal epithelial debridement model show the epithelium healing faster when treated with exosomes versus control [125] * Statistically significant.

Due to the known corneal wound-healing properties of MSC secretions, it was hypothesized that MSC exosomes could also have therapeutic properties. Samaeekia et al. studied the effect of corneal MSC exosomes on corneal epithelial wound healing [125]. The corneal MSCs were isolated from human cadaver corneas, with the secretome of the culture being collected. The exosomes were isolated using ultracentrifugation and stained for tracking. The stained exosomes were successfully absorbed by human corneal epithelium in vivo. Results on a scratch wound assay showed a marked improvement in corneal wound healing compared to the control group (Figure 4) [125]. Another study showed the anti-fibrotic effect of exosomes derived from L-MSCs. In a corneal epithelial debridement mouse model, Shojaati et al. demonstrated decreased scar formation with reduced expression of fibrotic markers in eyes treated with L-MSC-derived exosomes [126]. They also examined neutrophil infiltration and found that these exosomes reduced myeloperoxidase after 24 h, also indicating an anti-inflammatory effect. This demonstrates the potential of MSC exosomes as an option for corneal healing for patients with ocular surface injuries and LSCD.

More recently, the role of exosomes in immunomodulation has been studied. MSCs and other corneal exosomes have anti-inflammatory and immunosuppressive properties [127]. As the pathology of the limbal niche involves many inflammatory mediators, the immune-modulating properties of exosomes could be highly effective in treating LSCD and autoimmune eye diseases and stimulating corneal regeneration. This concept was demonstrated by Knickelbein et al., who studied exosomes from retinal pigment epithelial cells in the context of non-infectious uveitis. It was found that the released exosomes suppressed proliferation of T lymphocytes; this could be useful in therapies for reducing inflammation in uveitis [128]. Another study by Shigemoto-Kuroda et al. examined the use of MSC exosomes as a form of therapy for autoimmune uveoretinitis. They also found in a mixed lymphocyte reaction assay that MSC exosomes had an inhibitory effect on T cell proliferation as well as Th1 and Th17 lymphocyte development [129].

In the context of LSCD, exosomes are an attractive option to limit the progression of inflammation-mediated disease and so could be considered early in disease course before transplantation. However, there are a few disadvantages of using MSC-derived exosomes as a form of therapy in LSCD. The isolation and purification of these vesicles is not uniform across different studies, and, thus, advantages seen using a single isolation method might not be present in other populations of MSC-derived exosomes [130]. One study showed that varying the conditions under which exosomes are isolated led to a significant difference in the expression of pro-inflammatory cytokines within the different exosome populations [131]. Exosomes’ ability to modulate inflammation should be further explored in the context of LSCD; however, investigators should rigorously adhere to standardized isolation techniques to derive a homogenous population of vesicles.

Another benefit of exosomes is their use as a delivery vehicle, with the ability to load specific cargo. This was proven in the study conducted by Shtam et al., where exogenous siRNA was introduced into exosomes and delivered in vitro to cells [132]. One study applied this method within the context of retinal inflammation. Zhang et al. used MSC-derived exosomes loaded with exogenous microRNA (miRNA)-126 and injected into hyperglycemic rats with retinal inflammation. They found that administration of the exosomes successfully delivered the miRNA and suppressed the pathway leading to retinal inflammation [133]. This demonstrates the potential use of exosomes for the delivery of drugs, RNA, or other molecules for corneal-related diseases.

## 4. Conclusions

Injury and inflammation can lead to the disruption of homeostasis in the limbal niche, a specialized microenvironment that promotes the proliferation and differentiation of limbal epithelial stem cells. To restore the normal corneal structure and function, both replenishment of LESCs and long-term rehabilitation of the limbus are needed. Limbal transplantation and factor-derived therapy are examples of techniques being used clinically to treat this problem. Emerging approaches include epithelial- and mesenchymal-based cell therapies, new methods for synthetic scaffolds and new combinations of soluble factor therapies from cell secretions such as exosomes. These methods can accelerate corneal regeneration with potential application in limbal stem cell deficiency. While current results show promise for the future treatment of ocular surface diseases, in vivo studies and clinical trials are needed to ensure clinical safety and efficacy.
